# Handwriting movements for assessment of motor symptoms in schizophrenia spectrum disorders and bipolar disorder

**DOI:** 10.1371/journal.pone.0213657

**Published:** 2019-03-14

**Authors:** Yasmina Crespo, Antonio Ibañez, María Felipa Soriano, Sergio Iglesias, Jose Ignacio Aznarte

**Affiliations:** 1 Psychology Department, University of Jaén, Jaén, Spain; 2 Mental Health Unit, St. Agustín Universitary Hospital, Linares, Jaén, Spain; University of Toronto, CANADA

## Abstract

The main aim of the present study was to explore the value of several measures of handwriting in the study of motor abnormalities in patients with bipolar or psychotic disorders. 54 adult participants with a schizophrenia spectrum disorder or bipolar disorder and 44 matched healthy controls, participated in the study. Participants were asked to copy a handwriting pattern consisting of four loops, with an inking pen on a digitizing tablet. We collected a number of classical, non-linear and geometrical measures of handwriting. The handwriting of patients was characterized by a significant decrease in velocity and acceleration and an increase in the length, disfluency and pressure with respect to controls. Concerning non-linear measures, we found significant differences between patients and controls in the Sample Entropy of velocity and pressure, Lempel-Ziv of velocity and pressure, and Higuchi Fractal Dimension of pressure. Finally, Lacunarity, a measure of geometrical heterogeneity, was significantly greater in handwriting patterns from patients than from controls. We did not find differences in any handwriting measure on function of the specific diagnosis or the antipsychotic dose. Results indicate that participants with a schizophrenia spectrum disorder or bipolar disorder exhibit significant motor impairments and that these impairments can be readily quantified using measures of handwriting movements. Besides, they suggest that motor abnormalities are a core feature of several mental disorders and they seem to be unrelated to the pharmacological treatment.

## Introduction

Motor abnormalities (MA) are a relevant feature of several mental disorders [1]. MA have been widely studied in schizophrenia, from early descriptions of the disorder [2]. Later, with the discovery of antipsychotic drugs, MA were mainly studied as side effects of the pharmacological treatment. Nevertheless, recent research has shown a renewed interest in the study of MA in schizophrenia. It has been suggested that spontaneous and medication- independent motor phenomena can represent a specific dimension within the schizophrenia-spectrum [3]. MA have been detected in antipsychotic naïve patients with a first psychotic episode and even in individuals at high risk of psychosis [4–7]. Furthermore, MA have been observed in studies with children who later developed schizophrenia [8–12], as well as in chronic patients who had never been medicated [13]. These results seem to imply that MA have a central role in the prognosis and evolution of the disease and it has been signaled that they could facilitate accurate early detection and tailored intervention [14]. MA have been less studied in bipolar disorder. However, a wide amount of research has highlighted the commonalities between schizophrenia spectrum disorders and bipolar disorder [15–19]. Both disorders share genetic liability and some clinical features [20]. It has been shown that cognitive deficits and functioning is similar in early-onset schizophrenia and early-onset bipolar disorder, suggesting that cognitive dysfunction is more related to the neurodevelopmental course of the disorder than to the specific diagnosis [21].

Joined together, these results have led to a debate about whether schizophrenia spectrum disorders and bipolar disorders belong to different diagnostic categories, or to a common psychotic-affective spectrum [22]. Recent research has shown that certain cognitive and biological measures allow for better characterization of subtypes of patients with schizophrenia, schizoaffective and bipolar disorders than classical diagnosis methods [23]. MA could be a core characteristic in disorders within this spectrum. However, as we mentioned before, few studies have explored motor symptoms in bipolar disorder. For example, [24] found that patients with bipolar disorder performed worse on some measures of motor function [force steadiness and velocity scaling] than healthy participants. Furthermore, in a study from [25], with elderly people suffering from bipolar disorder, this group showed higher prevalence and increased severity of extrapyramidal symptoms [measured with observer-based rating scales] than controls. These findings were not associated with duration of illness or with current pharmacological exposure. More interestingly, [26] assessed motor performance in children of 7 years of age of parents with schizophrenia or bipolar disorder. They found that children of parents with schizophrenia showed significantly impaired motor performance compared to control children. On the contrary, there were no significant differences between children of parents with bipolar disorder and control children. Motor performance in children at risk of bipolar disorder was somewhat intermediate between children at risk of schizophrenia and control children.

In short, MA could have an essential role in the diagnosis of schizophrenia spectrum disorders and bipolar disorders. The inclusion of a motor domain would allow a better understanding of psychopathology, and may also reveal important contributions to disease processes across diagnoses [3,27].

Traditionally, MA have been evaluated through observation scales such as Abnormal Involuntary Movement Scale (AIMS) or the Simpson Angus Extrapyramidal Side Effects Scale (SAS). Nevertheless, these tests have demonstrated an insufficient predictive value [28]. For this reason, other methods have been proposed such as the recording of handwriting movements [29–32]. Several studies have found that some features of handwriting may be an objective measure of MA and a useful complement to the clinical assessment of patients [33–38]. In this line of research, studies differ in the specific measures employed. Some authors have used kinematic writing measures such as velocity, acceleration, average normalized jerk [39] or fluency [36,40–42]. Further, pressure or in air-time measures, have also been used in the evaluation of motor symptoms [35,43]. Normally, in order to obtain all these measures, it is necessary to previously segment the handwriting patterns.

A novel approach in this field has been the study of non-linear features of handwriting. For example, [44] obtained a selection of non-linear measures from Archimedes´spiral drawing, a standard test on the diagnosis of essential tremor. Several entropy algorithms were evaluated, and results showed that these non-linear measures were useful in discriminating between patients diagnosed with essential tremor and controls.

Finally, there is another approach that deals with the analysis of handwriting, based on the geometry of the handwritten patterns. From this approach the heterogeneity of handwriting patterns has been measured with an estimation of its lacunarity [45]. This variable describes the distribution of points and gaps in a geometric space [46–49] and it seems to be suitable for the analysis of handwritten texts. Indeed, it has been shown that the spatial heterogeneity of handwriting texts is sensitive to variations in cognitive demands of the handwriting task [45]. This analysis of handwritten patterns presents some advantages over kinematic or mechanical instruments: technical equipment is not required, and analyses can be applied even to past handwritten documents. In addition, it is not necessary to segment the texts. These advantages facilitate their application both in research and clinical settings.

In this research work, we aimed to examine the value of several measures of handwriting in the study of MA in individuals with a diagnosis of schizophrenia spectrum disorders and individuals with a diagnosis of bipolar disorder. We have collected classical, non-linear and geometrical measures of handwriting.

## Materials and methods

### Participants

54 adult individuals attending the Mental Health Day Unit at the University St. Agustin Hospital (Spain) participated in the study. Inclusion criteria were ICD-10 diagnosis of schizophrenia (F20), psychotic disorder (F23), schizoaffective disorder (F25) or bipolar disorder (F31), and age between 19 and 65 years old (*M* = 38.23; *SD* = 11.84). Diagnosis of participants was made using a semi-structured interview (SCID-I) according to ICD-10 criteria by the psychiatrist or clinical psychologist in charge of the patient. Out of the 54 participants, 16 (29.6%) were female. 53 participants were right-handed whereas 1 was left-handed. Their mean illness duration was 15.11 years (*SD* = 10.60). Regarding educational level, 1 had no formal studies, 15 had Primary education, 15 participants had Secondary education, and 8 had Higher education. Half of the patients (24) were taking different combinations of atypical antipsychotics (23), together with antidepressants (15), mood stabilizers (15), or typical antipsychotics (2), whereas 25 were only on atypical antipsychotics, 4 were only on antidepressants and 1 was unmedicated. There were no patients who suffered from Tardive Dyskinesia: all patients had absent or minimal symptomatology (a score of 0 or 1 in the items of the AIMS).

For the control group, 44 adults were recruited from the University of Jaén and an adult school of Jaén. The inclusion criterion was age between 19 and 65 years (*M* = 42.86 years old; *SD* = 14.47 years old). Out of the 44 participants, 26 were female. 41 participants were right-handed whereas 3 were left-handed. Regarding educational level, 2 participants had no formal studies, 16 had Primary education, 11 participants had Secondary education, and 3 participants had Higher education. There were no significant differences between groups on age (*t* = 1.73, *p* = 0.09) and educational level (*χ*^2^ = 2.59, *p* = 0.62).

Exclusion criteria for both groups were: concurrent diagnosis of neurological disorder, concurrent diagnosis of substance abuse, history of developmental disability, inability to sign informed consent or vision disorders (those vision disorders which, although corrected by glasses or contact lenses, suppose a loss of visual acuity, e.g., cataracts). In addition, an exclusion criterion for the control group was the diagnosis of a mental disorder (according to verbal reports from participants).

All participants gave their written informed consent according to the Declaration of Helsinki and the Ethics Committee on Human Research of the Hospital approved the study.

### Materials

The patients group were evaluated with the Simpson-Angus Scale (SAS) and the Positive and Negative Syndrome Scale (PNSS).

The SAS is a rating scale used for the assessment of drug-induced parkinsonism in both clinical practice and research settings [50]. The scale is composed of ten items. It consists of one item measuring gait (hypokinesia), six items measuring rigidity and three items measuring glabella tap, tremor and salivation, respectively. For each item, severity of symptoms is rated from 0 (none) to 4 (severe). Although SAS scores can range from 0 to 40, a mean global score of 3 or more is used as a threshold to indicate the presence of the extrapyramidal symptoms in a mild form [51]. In our sample, the Cronbach alpha was 0.83, and the mean score was 5.65 points (*SD* = 4.77 points). The highest score was 21 points.

The Spanish version [52] of the Positive and Negative Syndrome Scale; [53,54] was also used to evaluate to the participants. The PANSS is a rating scale that is commonly employed to measure the severity of psychotic symptoms [53,54]. It can be divided in three subscales: the positive subscale (PANSS-P, alpha = 0.76) of 7 items (*M* = 14, *SD* = 6.08), the negative subscale (PANSS-N, alpha = 0.89) of 7 items (*M* = 18.85, *SD* = 7.60), and the general psychopathology subscale (PANSS-PG, alpha = 0.83) of 16 items (*M* = 32.41, *SD* = 9.33).

### Procedure

**Fig 1:**
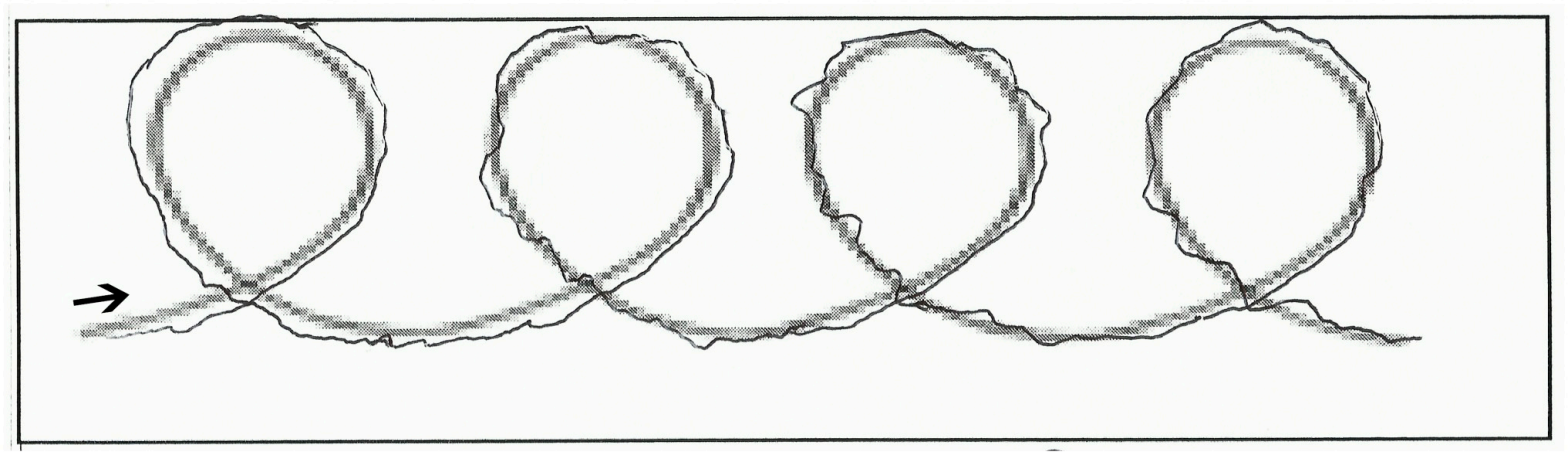
Handwriting task.

Participants were asked to perform an easy and brief handwriting task. An A4 paper was affixed to the surface of a WACOM (Intuos pro small) digitizing tablet. A four loops model was presented on the paper, and participants were required to write four loops using this template, with a wireless inking electronic pen (Fig 1). After that, a clinician administered the PANSS and SAS scales, and some sociodemographic data (age, gender, educational level) were collected. Handwriting tasks were carried out individually. The task had no time limit.

### Measures of handwriting

Handwriting data were recorded at a sampling frequency of 200 Hz and a spatial resolution of.05 mm. Handwriting measures were first obtained through Ductus software and then derived through Matlab. Ductus software is a tool designed to analyze and aid in the understanding of processes underlying handwriting production [55]. Different types of measures were included:

#### Classical measures

Velocity, acceleration, length, number of peaks and pressure were measures derived from the Ductus software using Matlab. The recorded X and Y position data were smoothed with a low pass filter with normalized cut-off frequency. Mean velocity was calculated by averaging the absolute velocity values per time and position in each participant (in cm/sec). Mean acceleration was calculated by averaging the absolute acceleration (in cm^2^/sec) in each participant. Length trajectory was the total path (in cm) of the pen on the surface of the digitizing tablet for the four loops. Movements disfluency was measured with the number of absolute velocity peaks of each participants in the four loops [56]. Smooth movements produce less velocity peaks than disfluent movements, and pressure refers to the pressure of the pen on the digitizing surface (in nonscale units).

#### Non-lineal measures

Higuchi fractal dimension (HFD), Sample entropy (SE), and Lempel-Ziv (LZ) measures were used as signal complexity estimators for velocity and handwriting pressure. Complexity measures capture the degree of randomness in time series. HFD is a measure of the self-similarity of the signal. HFD take values between 1 (simple curves), and 2 (random signals) [57]. SE is a measure of irregularity based on the conditional probability that subseries of the signal that match at each point within a certain tolerance also match at the next point [58]. LZ is a measure that computes the number of different substrings in a signal and its rate of recurrence [59]. The goal in this study was to obtain features in the signal that could help in identifying diagnostic elements of the handwritings. All of these measures have been successfully applied in neurophysiology research of severe mental illness [60–62].

#### Geometrical measures: Lacunarity

To characterize the geometrical structure of the handwriting we used Lacunarity, which is a specific measure of geometrical invariance. Lacunarity is an estimator of structural homogeneity, and measures the density of points (the proportion of filled compared to empty pixels) and the clumping of points and gaps [63]. Hence, to obtain this measure, we needed to consider the handwriting patterns as 2D images where the time variable was irrelevant. All handwriting patterns were scanned with a resolution of 900 ppi, and size of 2402x1801 ppp. Then grey images were converted into binary. Lacunarity was then calculated using the algorithm from [47] (see the exact computations in [45]).

### Data analysis

Handwriting measures were compared between groups (Patients vs Controls) using a t-test for independent samples. In those cases in which Levene’s test for homogeneity of variances indicated that there was a significant difference in the size of within variances, we used the alternative version of *t*-test for unequal variances [64]. In those cases in which the assumption of normality was not met, data were log-transformed. When transformation was not successful in order to normalize the distributions, we used the nonparametric Mann-Whitney U test.

To study whether handwriting variables could have been affected by psychopathology (PANSS) or whether SAS scores could be influenced by psychopathology (PANSS) Spearman rank order correlations were computed.

In addition, in order to explore the possibility that differences in diagnosis could contribute to subgroup effects on the handwriting tasks, we conducted *t*-test in order to compare both groups (Schizophrenia vs Bipolar disorder) in the handwriting variables. Finally, we classified patients depending on antipsychotics doses, and we conducted *t*-test in order to explore whether antipsychotic treatment influenced handwriting measures.

## Results

### Classical measures

The results for the kinematic variables are shown below: Velocity, Acceleration, Length, number of Peaks and Pressure. See Table 1 for a summary.

**Table 1:** Kinematic variables and their mean values and standard deviations (between parenthesis) for each group (Patients vs Controls). Parametric and non-parametric test results are shown under Student’s t and Mann-Whitney’s U respectively.

Variables	Patients (N = 54)	Healthy Comparison Subjects (N = 44)	Student’s t
Velocity	2.24 (0.80)	3.20 (1.30)	*t* = -4.25, *p*<0.001
Acceleration	0,04 (0.60)	0,11 (0.13)	*t* = -3.37, *p*<0.001
Length	27.57 (17.47)	17.19 (7.03)	*t* = 3.78, *p*<0.001
Peaks	241.73 (134.21)	160.45 (64.39)	*t* = 3.49, *p*<0.001
Pressure	755.30 (153.48)	664.76 (185.27)	*t* = 2.64, *p* = 0.01

**Fig 2:**
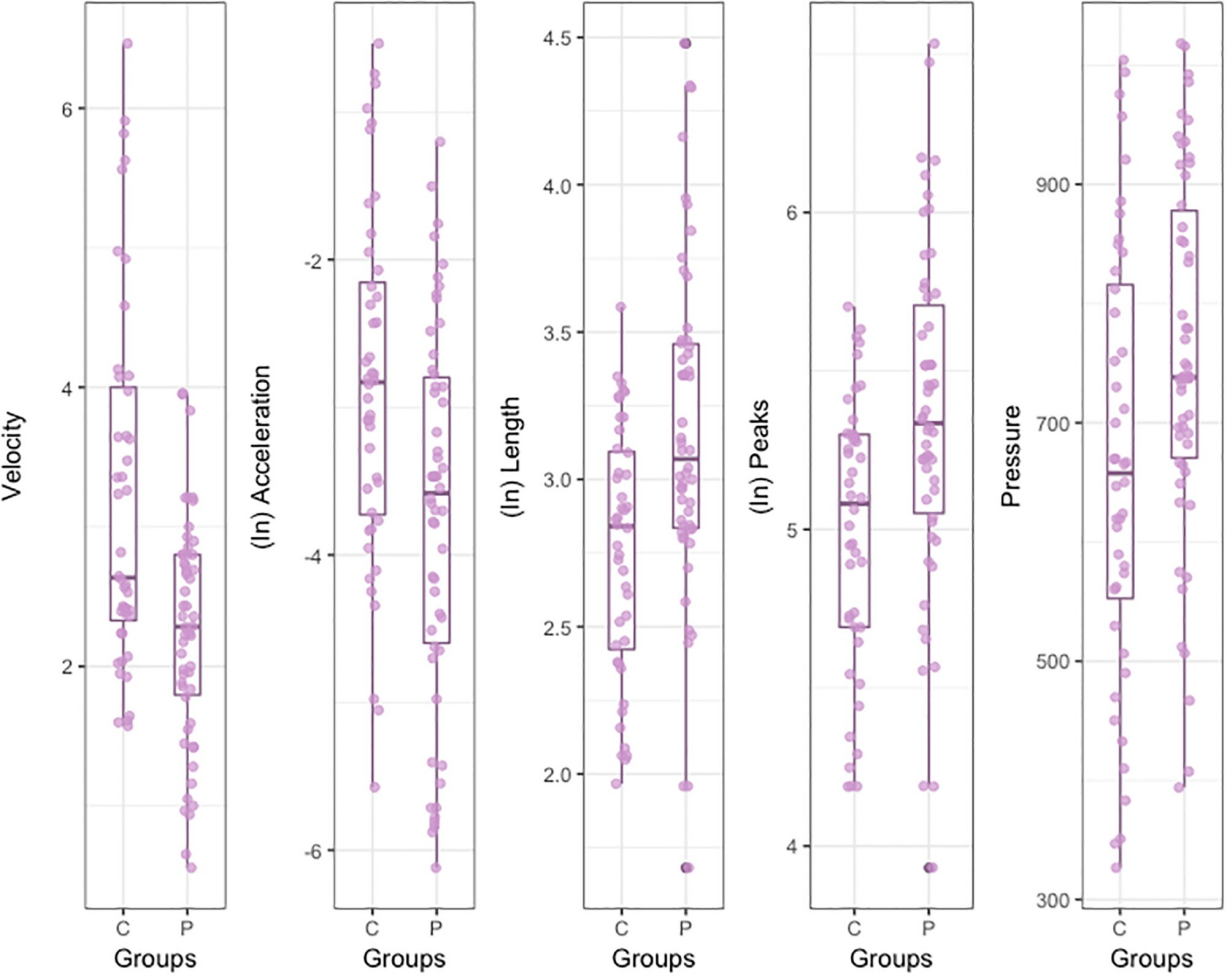
Box Plot for handwriting variables.

Results obtained with *t*-test for unequal variances [64] for *Velocity* showed that the handwriting of the Patients was significantly slower than that of the Controls (Table 1 and Fig 2).

Due to the *Acceleration* distributions for both groups were positively skewed, a log-transformation was conducted in order to symmetrize the data. Shapiro-Wilk test showed that transformations were successful [Patients: *W* = 0.96, *p* = 0.13; Controls: *W* = 0.98, *p* = 0.89]. Results in *Acceleration* indicated that patients showed a significant lower mean than Controls (Fig 2).

Due to the skewness in *Length*, a log-transformation was conducted [Patients: *W* = 0.97, *p* = 0.22; Controls: *W* = 0.96, *p* = 0.15]. Results indicated that Patients showed a significant higher mean in (log-transformed) *Length* than Controls (Fig 2).

As with the previous variables, it was necessary to transform the number of *Peaks* of velocity to achieve normality in the distributions [Patients: *W* = 0.98, *p* = 0.73; Controls: *W* = 0.95, *p* = 0.05]. Results indicated that Patients showed a significant higher average number of *Peaks* than Controls (Fig 2).

Regarding *Pressure*, results indicated that Patients showed a significant higher *Pressure* than Controls (Fig 2).

### Complexity measures

The results for complexity variables are shown below: HFDv (velocity HFD), SEv, (velocity SE), LZv (velocity LZ), HFDp (pressure HFD), SEp (pressure SE), and LZp (pressure LZ). See Table 2 for a summary.

**Table 2:** Complexity variables and their mean values and standard deviations (between parenthesis) for each group (Patients vs Controls). Parametric and non-parametric test results are shown under Student’s t and Mann-Whitney’s U respectively.

Variables	Patients (N = 54)	Healthy Comparison Subjects (N = 44)	Student’s t	Mann-Whitney’s U
HFDv	1.54 (0.06)	1.54 (0.07)	*t* < 1	
SEv	0.70 (0.19)	0.60 (0.18)	*t* = 2.57, *p* = 0.01	
LZv	0.39 (0.06)	0.36 (0.06)	*t* = 2.03, *p* = 0.04	
HFDp	1.24 (0.06)	1.20 (0.04)	*t* = 3.23, *p*<0.01	
Sep	0.039 (0.01)	0.046 (0.01)	*t* = -2.42, *p*<0.01	
LZp	0.056 (0.02)	0.064 (0.002)		*z* = 2.22, *p* = 0.02
Lacunarity	0.27 (0.02)	0.26 (0.02)		*z* = -2.11, *p* = 0.03

**Fig 3:**
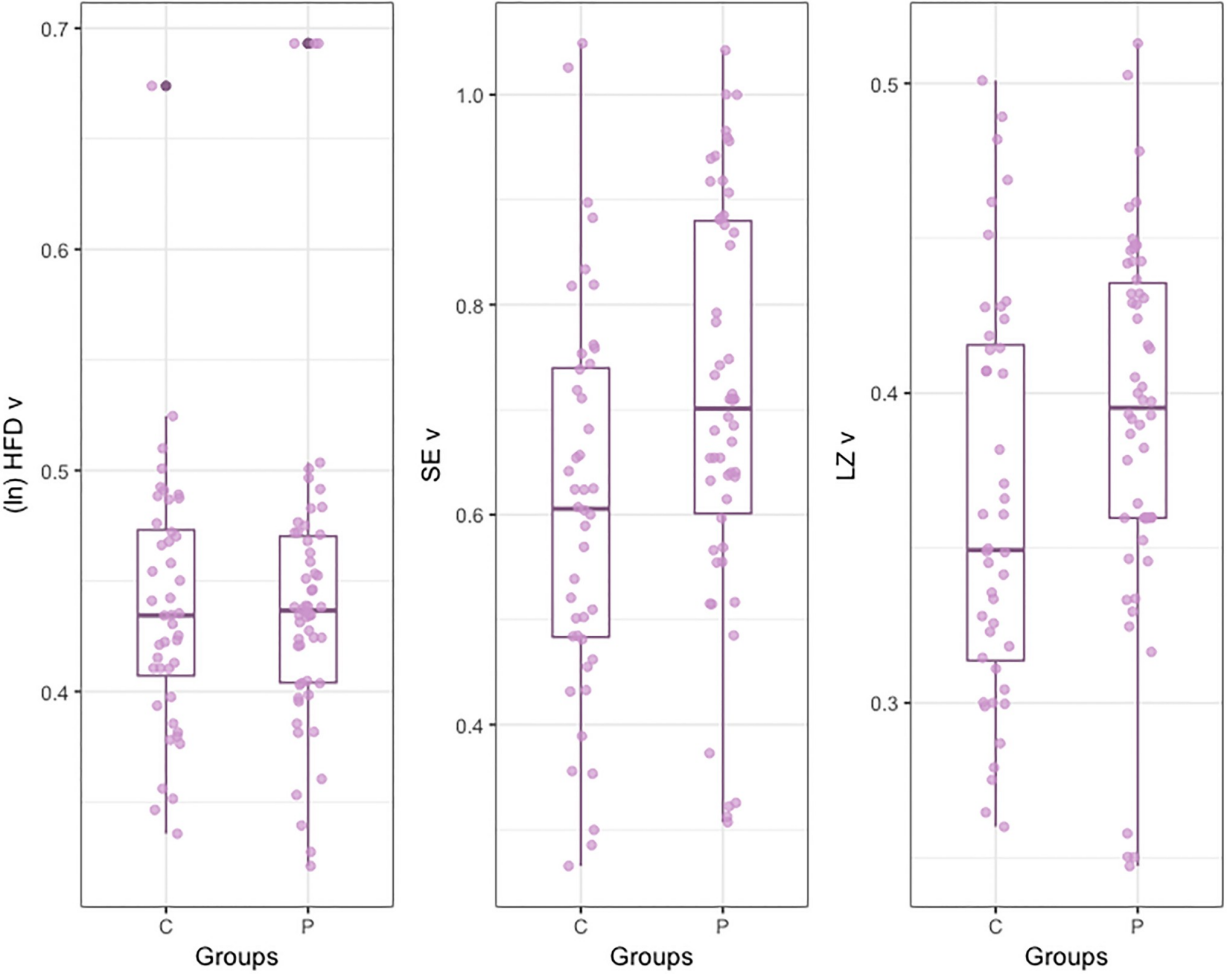
Box Plot for complexity measures for velocity.

It was necessary to transform the *HFDv* to achieve normality in the distributions [Patients: *W* = 0.96, *p* = 0.09; Controls: *W* = 0.98, *p* = 0.66]. Results indicated that differences in *HFDv* between groups were not significant (Fig 3). Regarding *Sev*, results indicated that *SEv* in Patients was significantly higher than in Controls. Similarly, *LZv* in Patients was significantly higher than in Controls (Fig 3).

**Fig 4:**
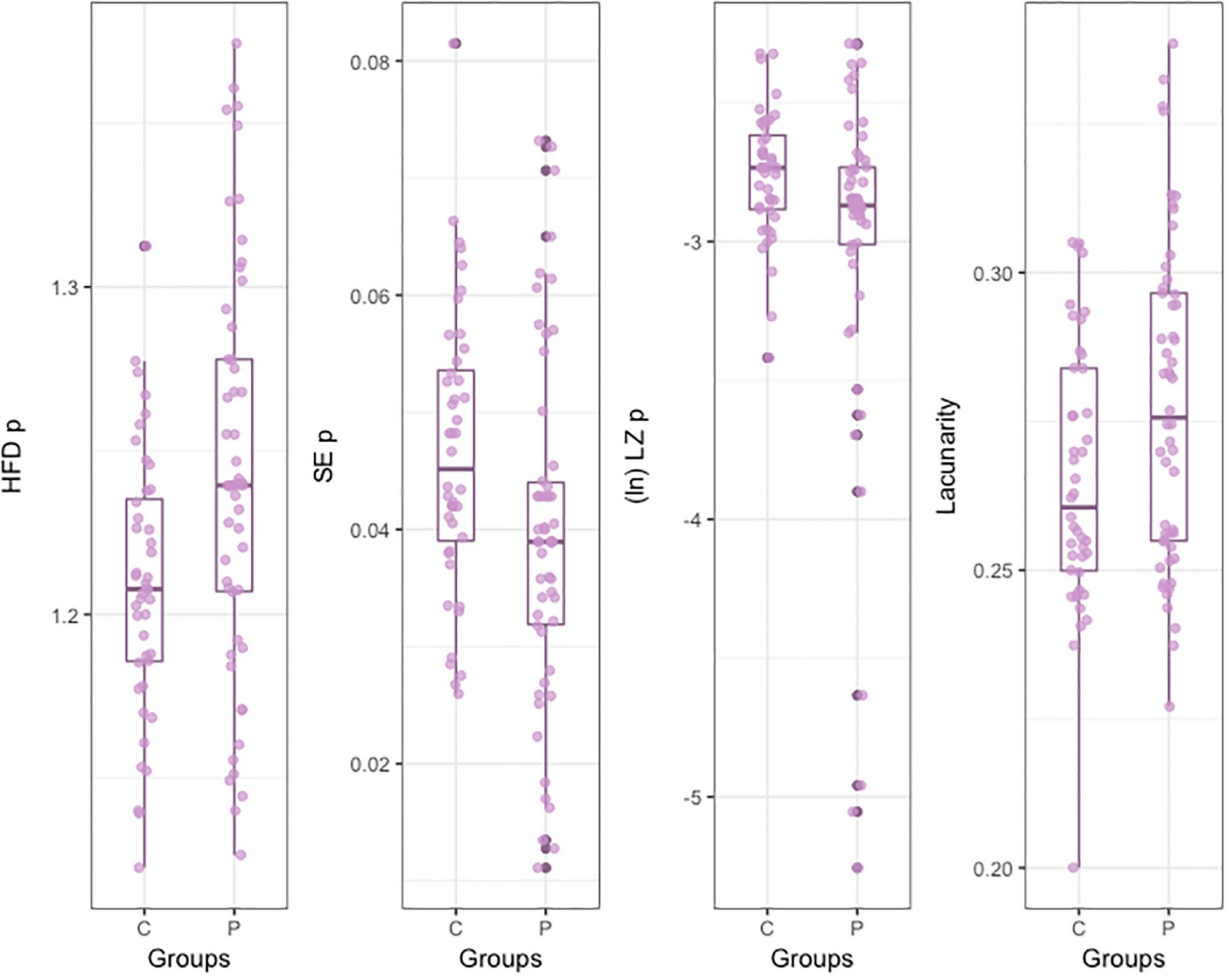
Box Plot for complexity measures for pressure and lacunarity.

Results with *t*-test for unequal variances indicated that the *HFDp* of the Patients handwritings was significantly higher than that of the Controls. On the contrary, the *SEp* of the Patients handwritings was significantly lower than that of the Controls (Fig 4).

Shapiro-Wilk test showed that *LZp* distributions were not normal [Patients: *W* = 0.93, *p*<0.001; Controls: *W* = 0.97, *p* = 0.38]. Log transformations were carried out in order to symmetrize data distributions, but transformed distributions remained not-normal. Due to the abnormality of data, Mann-Whitney test was conducted on untransformed data, and it indicated that *LZp* was significantly lower for Patients than for Controls (Fig 4).

Finally, Shapiro-Wilk test showed that *Lacunarity* distributions were not normal [Patients: *W* = 0.96, *p*<0.12; Controls: *W* = 0.92, *p*<0.01]. Log transformations were carried out in order to symmetrize data distributions, but transformed distributions remained not-normal. Mann-Whitney test was conducted on untransformed data, indicating that *Lacunarity* was significantly higher for Patients than for Controls (Fig 4).

### Relation between movement abnormalities and psychopathology

**Fig 5:**
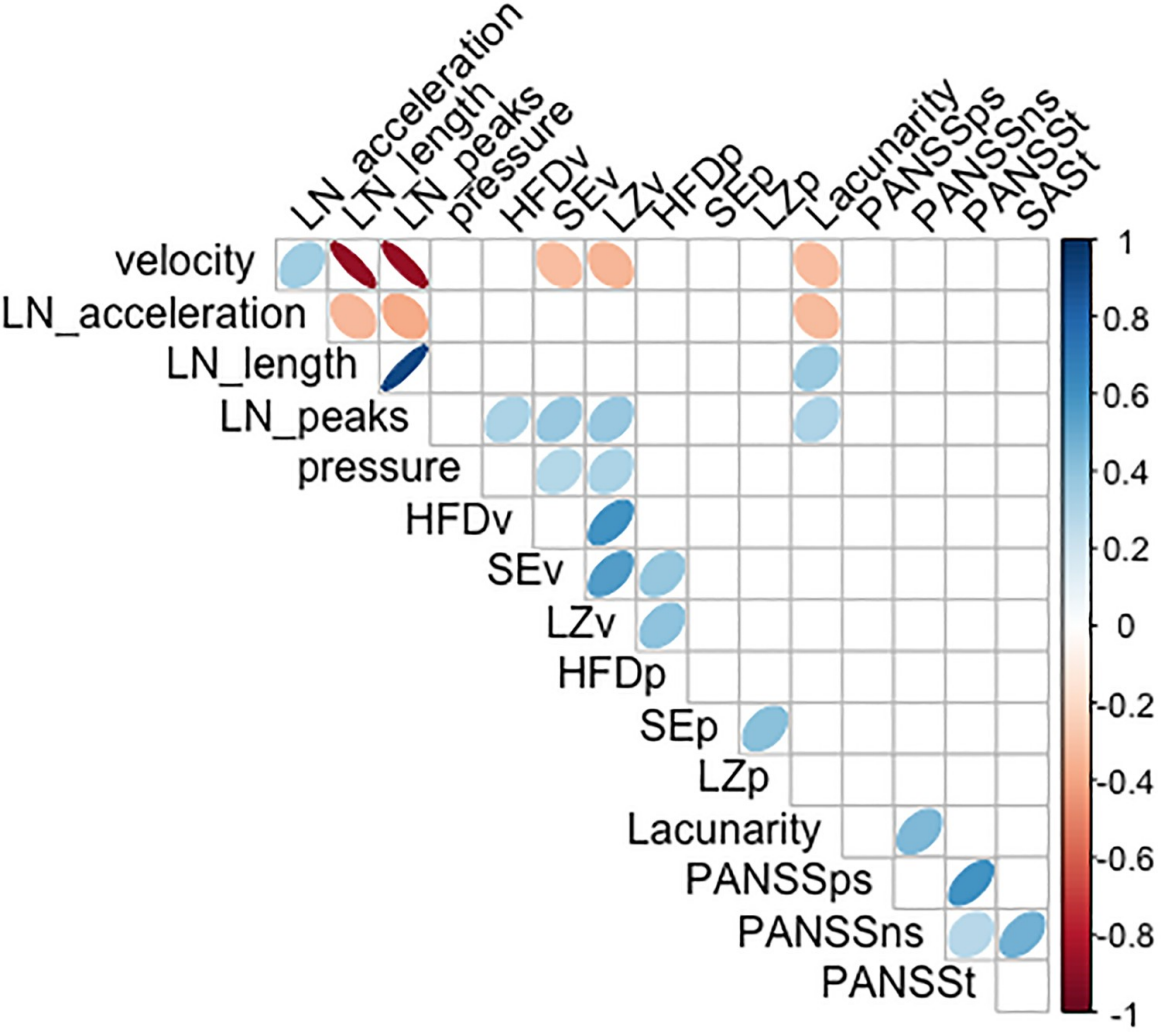
Spearman coefficients for SAS, PANSS and the handwriting variables.

To study whether handwriting task performance could have been affected by psychopathology (PANSS) or whether SAS scores could be influenced by psychopathology (PANSS) correlational analyses were performed (Fig 5). Specifically, correlations were computed between SAS, PANSS and the handwriting variables. Due to non-normal distribution of some variables Spearman rank order correlation was computed.

We found that Severity of EPS (based on SAS total score) was associated with PANSS negative symptom severity (*r*s = 0.47, *p*<0.01). Out of handwriting measures, only Lacunarity was significantly associated with PANSS negative symptom severity (*r*s = 0.44, *p*<0.01).

### Effects of Diagnosis: Schizophrenia vs bipolar disorder

In order to explore the possibility that differences in diagnosis could contribute to subgroup effects on the handwriting tasks, we compared both groups (Schizophrenia vs Bipolar disorder) in the handwriting variables. According to Shapiro-Wilk test results, all variables were normally distributed for both groups, consequently, *t*-test were conducted. For all comparisons Levene’s test showed homogeneity of variances. Levene and Shapiro-Wilk results are not reported. No significant differences were found between Schizophrenia and Bipolar disorder patients for any of the handwriting variables. Student’s *t* test results are summarized in Table 3.

**Table 3:** Handwriting variables and their mean values and standard deviations (between parenthesis) for each group (Schizophrenia vs Bipolar disorder).

Variables	Schizophrenia spectrum disorder (N = 43)	Bipolar Disorder (N = 11)	Student’s t
Velocity	2.20 (0.80)	2.41 (0.80)	*t*<1, *p* = 0.46
Acceleration	637.95 (629.51)	756.61 (907.28)	*t*<1, *p* = 0.67
Length	28.35 (18.37)	24.52 (13.73)	*t*<1, *p* = 0.47
Peaks	249.31 (141.28)	212.09 (102.05)	*t*<1, *p* = 0.44
Pressure	756.76 (159.16)	749.61 (135.67)	*t*<1, *p* = 0.89
HFDv	1,53 (0.06)	1,56 (0.04)	*t* = -1.59, *p* = 0.11
SEv	0,68 (0.19)	0,77 (0.14)	*t* = -1.35, *p* = 0.18
LZv	0,38 (0.06)	0,41 (0.03)	*t* = -1.52, *p* = 0.13
HFDp	1,23 (0.06)	1,25 (0.06)	*t*<1, *p* = 0.47
SEp	0,03 (0.01)	0,04 (0.01)	*t*<1, *p* = 0.91
LZp	0,05 (0.02)	0,05 (0.01)	*t*<1, *p* = 0.73
Lacunarity	0,27 (0.02)	0,27 (0.02)	*t*<1, *p* = 0.41
SAS	5.72 (4.90)	5.36 (4.41)	t<1*p* = 0.82

In order to explore whether results were influenced by age or educational level, ANCOVAs were performed on Handwriting measures controlling for Age and, Study level. We did not find significant results (all *p*s >.05).

### Medication effects

To study the putative effect of antipsychotic dose on the handwriting variables, medication dose was categorized as *high* or *low*, and *t*-tests were conducted on each handwriting variable. Categorization of treatments in *high* or *low* was based on clinical guidelines [65,66].

Shapiro-Wilk test showed that all variables were normally distributed for both groups (high vs low), consequently, *t*-test were conducted. When Levene test for homogeneity of variances was significant, alternative version of t-test for unequal variances [64] was conducted. Shapiro-Wilk test and Levene test are not reported. Results of Student’s *t* test results are summarized in Table 4

**Table 4:** Handwriting variables and their mean values and standard deviations (between parenthesis) for each group (Low vs High dose).

	Low dose (N = 29)	High Dose (N = 17)	Student’s t
Velocity	2.23 (0.84)	2.36 (0.83)	*t*<1
Acceleration	0.03 (0.04)	0.06 (0.08)	*t* = -1.06, *p* = 0.29 a
Length	26.46 (18.13)	25.58 (16.65)	*t*<1
Peaks	243.39 (152.68)	225.62 (120.90)	*t*<1
Pressure	728.92 (161.73)	781.44 (148.30)	*t =* -1.09, *p* = 0.27
HFDv	1.53 (0.06)	1.54 (0.05)	*t*<1
SEv	0.69 (0.20)	0.70(0.20)	*t*<1
LZv	0.38 (0.06)	0.39 (0.04)	*t*<1
HFDp	1.24 (0.06)	1.21 (0.04)	*t =* -1.62, *p* = 0.09
SEp	0.03 (0.02)	0.06 (0.01)	*t*<1
LZp	0.05 (0.02)	0.04 (0.02)	*t* = 1.54, *p* = 0.13
Lacunarity	0.27 (0.02)	0.27 (0.02)	*t*<1
SAS	5.00 (5.23)	6.58 (4.79)	*t* = 1.02. *p* = 0.31

^a.^ Due to non-homogeneity of variances alternative version of *t*-test for unequal variances was conducted [64].

Moreover, in order to explore the effect of antidepressants on our handwriting measures, participants were classified in two groups depending on whether they were receiving or not antidepressants. Shapiro-Wilk test showed that all variables were normally distributed for both groups; consequently, *t*-test were conducted. No significant differences between groups were observed in none of the handwriting measures. Results of Student’s *t* test results are summarized in Table 5.

**Table 5:** Complexity variables and their mean values and standard deviations (between parenthesis) for each group (No antidepressant vs Antidepressant). Parametric test results are shown under Student’s *t*.

	No antidepressant (N = 35)	Antidepressant (N = 19)	Student’s *t*
Velocity	5.71 (4.92)	5.53 (4.60)	*t* < 1
Acceleration	0.04 (0.06)	0.05 (0.05)	*t* < 1
Length	26.53 (15.28)	29.48 (21.26)	*t* < 1
Peaks	232.58 (106.18)	258.64 (176.84)	*t* < 1
Pressure	752.08 (151.32)	761.26 (161.42)	*t* < 1
HFDv	1.54 (0.06)	1.55 (0.07)	*t* < 1
SEv	0.73 (0.18)	0.67 (0.20)	*t* = 1.07. *p* = 0.28
LZv	0.39 (0.06)	0.39 (0.06)	*t* < 1
HFDp	1.25 (0.06)	1.24 (0.06)	*t* < 1
Sep	0.04 (0.01)	0.04 (0.02)	*t* < 1
LZp	0.06 (0.02)	0.05 (0.02)	*t* < 1
Lacunarity	0.28 (0.02)	0.27 (0.02)	*t* = 1.58. *p* = 0.12

## Discussion

Motor abnormalities are included among the diagnosis criteria of many mental disorders such as schizophrenia [67]. Motor abnormalities have an important implication for the etiology of schizophrenia [68,69]. However, they have been neglected in other mental disorder as bipolar disorder, although many features in common between schizophrenia and bipolar disorder have been pointed out [18,19]. In this line, both disorders are genetically related [23,70] and have overlapping clinical phenomenology [71,72].

One of the main problems in the research about MA is the lack of objective and reliable measuring tools [73]. Rating scales can be considered as the most relevant instruments applied in clinical trials [74]. Nevertheless, these tests have demonstrated having an important lacks specificity [28]. But a fruitful line of research has focused on the evaluation of MA through writing. These studies have employed kinematic or non-lineal analysis of handwriting movements on a digitized tablet [44,75,76].

In the present study, our main aim was to explore the value of several measure of handwriting in the study of MA in patients with schizophrenia spectrum disorders or bipolar disorder.

Several important findings can be highlighted. First, that participants with a schizophrenia spectrum or bipolar disorder exhibit significant motor impairments that can be readily quantified using measures of handwriting movements recorded by a digitizing tablet. The handwriting of patients was characterized by a significant decrease in velocity and acceleration and an increase in length, number of peaks and pressure with respect to the handwriting of healthy controls. Thus, patients display a marked slowing of movements and a more disfluent handwriting than controls. These results converge with current evidence showing that handwriting disfluency is related to motor symptoms in psychotic disorders [39,75,77,78]. For example, [78] found that tardive dyskinesia patients exhibited significantly higher disfluency scores than non-tardive dyskinesia patients and controls.

Concerning the complexity measures, SEv, LZv, and HFDp of handwritings from patients were significantly higher than those from controls, while SEp and LZp were significantly lower for Patients than for Controls. As mentioned above, HFD, SE and LZ are measures that quantify the complexity in a signal in different ways. While HFD maps the self-similarity of the signals, LZ and SE are more closely related to entropy (entropy is a concept addressing randomness and predictability, with greater entropy often associated with more randomness and reduced order in the system). Thus, convergent results would be expected with both indicators (LZ and SE). The results indicate, in fact, a common pattern. On the one hand, the speed of the handwriting of patients turned out to be more complex than that of the controls, which would indicate a more irregular pattern in the handwriting of the patients, with more changes in speed, suggesting a reduction in motor control. Regarding pressure, the pattern is less complex in patients than in controls, which could be interpreted as a higher sustained pressure, a pattern that would not be characteristic of a fluid writing. The fact that HFD_p_ is higher in patients than controls (opposite pattern when compared with LZ_p_ and SE_p_) can be explained if the pressure in patients shows stereotypical changes that increase dimensionality and decrease randomness; that is, pressure time series exhibited more components in patients but they were more repetitive and predictable than pressure changes from controls. Similar discrepancies between Fractal Dimension measures and Entropy-based measures have been found in the analysis of reading fluency [79].

Finally, Lacunarity was significantly greater in the handwriting patterns from patients than controls. These results suggest a more heterogeneous handwriting patterns in patients than in controls, and this could be related to the disfluency of handwriting in patients (greater number of peaks of velocity).

A second important finding is that we did not find significant differences between participants of function of the diagnosis (schizophrenia spectrum disorder or bipolar disorder) in any of the handwriting measures evaluated nor in SAS scores. These results support research which has highlighted the commonalities between schizophrenia and bipolar disorder [18] and they suggest that schizophrenia spectrum disorders and bipolar disorder could be part of the same clinical spectrum [22].

And third, we did not find significant differences between participants with low and high doses of antipsychotics in any of the handwriting measures evaluated. These results converge with other studies that detected MA in antipsychotic naïve patients with a first psychotic episode [6], or even in individuals at high risk of psychosis who have never been in pharmacological treatment [39,80]. In addition, results are in accordance with recent views that pharmacological treatment may not only worsen, but can also left unchanged or even improve MA [1]. However, it should be noted that in our study there are no patients without medication. In clinical practice, it is difficult to find individuals with a severe mental disorder who are not under the effect of medication. Hence, it is difficult to assert whether motor symptoms as captured by heterogeneity of handwriting are related to the disorder or to the pharmacological treatment. Future works could explore in depth this issue. We did not find differences between participants taking antidepressants and those who were not on antidepressants. These results seem to be in contradiction with some studies that show handwriting abnormalities in people under the effects of tricyclic antidepressant drugs. Specifically, [81] found that individuals receiving tricyclic antidepressants, in comparison with both healthy subjects and patients receiving serotonin re-uptake inhibitors displayed an increased movement time, reduced automation of handwriting, lower maximum velocities and reduced acceleration of descending strokes. However, in our study, 17 patients were taking serotonin re-uptake inhibitors and only two were taking tricyclic antidepressants. On the other hand, the two participants receiving tricyclic antidepressants in our study were treated with much lower doses (average dose of 37.5 mg) than in the study by [81] (average dose of 125 mg).

Taken together, these results demonstrated that MA affect handwritten patterns. MA have been related to dysfunctions in the connectivity among the primary motor area, ventral premotor area, supplementary motor area, basal ganglia as well as cerebellum [82]. All these areas have an important role in the process of handwriting; particularly basal ganglia and the supplementary motor area have been shown to be involved in the planning and execution of hand movements in handwriting [83]. Thus, dysfunctions on these areas in certain mental and neurological disorders could provoke stiff and inflexible movements that would produce heterogeneous and irregular handwritten patterns.

Several limitations of the present research could be highlighted. First, as we mentioned before, all our patients were on antipsychotic treatment. Therefore, we can not clearly assert whether motor dysfunction is related to the disorder or to the pharmacological treatment. In addition, although it would have been really interesting to study the differential effect of typical antipsychotics versus atypical antipsychotics, this has not been possible. Nowadays, it is difficult to find patients on typical antipsychotics, precisely due to their known side effects. In our sample, only 2 patients were taking typical antipsychotics, and they were on a combination of typical and atypical antipsychotics. Therefore, it was not possible to isolate the effect of this variable. Future studies with larger and more heterogeneous samples could explore the effect of the specific type of medication on handwriting patterns. Second, we were not able to match the groups in gender (the control group had different female/male proportion than the patients groups). Female patients camouflage their difficulties better than males, and usually present less severe behavioral problems; hence, the wide majority of patients attending a Mental Health Day Hospital for severe mental disorders, are men. However, there is no evidence about a different profile of motor symptoms depending of gender; hence, we believe gender differences between groups is not influencing the results. Finally, psychotic symptoms were measured through the PANSS scale, which was specifically developed and validated for schizophrenia, and not for bipolar disorder. But it should be noted that the PANSS has been widely used in clinical trials to measure symptoms change in bipolar disorder [84,85] and recent research has shown that the factor structure of the scale is similar in schizophrenia and bipolar disorder [86].

The analysis of handwritten patterns presents some advantages over kinematic or mechanical instruments: technical equipment is not required, and analyses can be applied even to past handwritten patterns. These advantages facilitate their application both in research and clinical settings. In addition, with the extended use of computers and smartphones, there are new promising options for the measurement of motor dysfunctions (see, for example, the novel analysis of key presses proposed by [87] in the evaluation of psychomotor impairment). Writing movements, either handwriting or typing, can provide key information about motor symptoms, and can have relevant clinical applications.
